# ﻿*Hymenophyllumchamaecyparicola* (Hymenophyllaceae), a new filmy fern species from Taiwan

**DOI:** 10.3897/phytokeys.204.86045

**Published:** 2022-08-04

**Authors:** Zhi-Xiang Chang, Tian-Chuan Hsu, Li-Yaung Kuo

**Affiliations:** 1 Fushan Research Center, Taiwan Forestry Research Institute, No. 1, Fushan, Shuangpi Rd., Yilan 264013, Taiwan Taiwan Forestry Research Institute Yilan Taiwan; 2 Botanical Garden Division, Taiwan Forestry Research Institute, No. 53, Nanhai Rd., Taipei 10066, Taiwan Taiwan Forestry Research Institute Taipei Taiwan; 3 Department of Life Sciences, National Chung Hsing University, Taichung 402202, Taiwan National Chung Hsing University Taichung Taiwan; 4 Institute of Molecular & Cellular Biology, National Tsing Hua University, Hsinchu 30013, Taiwan National Tsing Hua University Hsinchu Taiwan

**Keywords:** Filmy fern, *Hymenophyllum*, new species, Taiwan

## Abstract

*Hymenophyllumchamaecyparicola* T.C.Hsu & Z.X.Chang, a new filmy fern species (Hymenophyllaceae) has been described from Taiwan and illustrated based on morphological and phylogenetic evidence. Although the new species resembles members in the subgenus Mecodium, namely *H.wrightii*, our plastid phylogeny has revealed that it is genetically distant from *H.wrightii* and forms a clade nested within subg. Hymenophyllum. The most notable characteristic to differentiate *H.chamaecyparicola* from related species is the presence of minute spathulate hairs on the surface of the rachis and veins. *Hymenophyllumchamaecyparicola* is currently only known from a small area in northern Taiwan, and endemic to that country.

## ﻿Introduction

Hymenophyllum is the largest subgenus among the ten subgenera in genus *Hymenophyllum* Sm., and includes at least 100 species (Ebihara et al. 2006; PPG I 2016). Generally, the members of this subgenus are distinguished by their long-creeping rhizomes, pinnate to tripinnate laminae, denticulation on the segment margins, bivalvate involucres, and included receptacles (Ebihara et al. 2006; Hennequin et al. 2010). However, subg. Hymenophyllum as a whole varies greatly in many aspects, including cytology and morphology (Hennequin et al. 2010). With a considerably large number of morphologically disparate species, members of subg. Hymenophyllum have been scattered among different groups or genera in Hymenophyllaceae (Pryer et al. 2001; Ebihara et al. 2002; Hennequin et al. 2003, 2006, 2010; Hennequin 2004) leaving many systematic issues in this subgenus unsettled. In Taiwan, 26 species are currently known to belong to the genus *Hymenophyllum*. Among them, eight were recognized in subg. Hymenophyllum (Hsu et al. 2019; TPG 2019, 2021), including *H.barbatum* (Bosch) Baker, *H.blandum* Racib., *H.denticulatum* Sw., *H.devolii* M.J.Lai, *H.holochilum* (Bosch) C.Chr., *H.okadae* Masam., *H.oligosorum* Makino and *H.simonsianum* Hook. Subgenus Hymenophyllum can be distinguished from its sister subgenus Mecodium by the presence of indumentum along the stipe, rachis, costae and veins vs. these surfaces glabrous in subgen. Mecodium.

**Figure 1.**
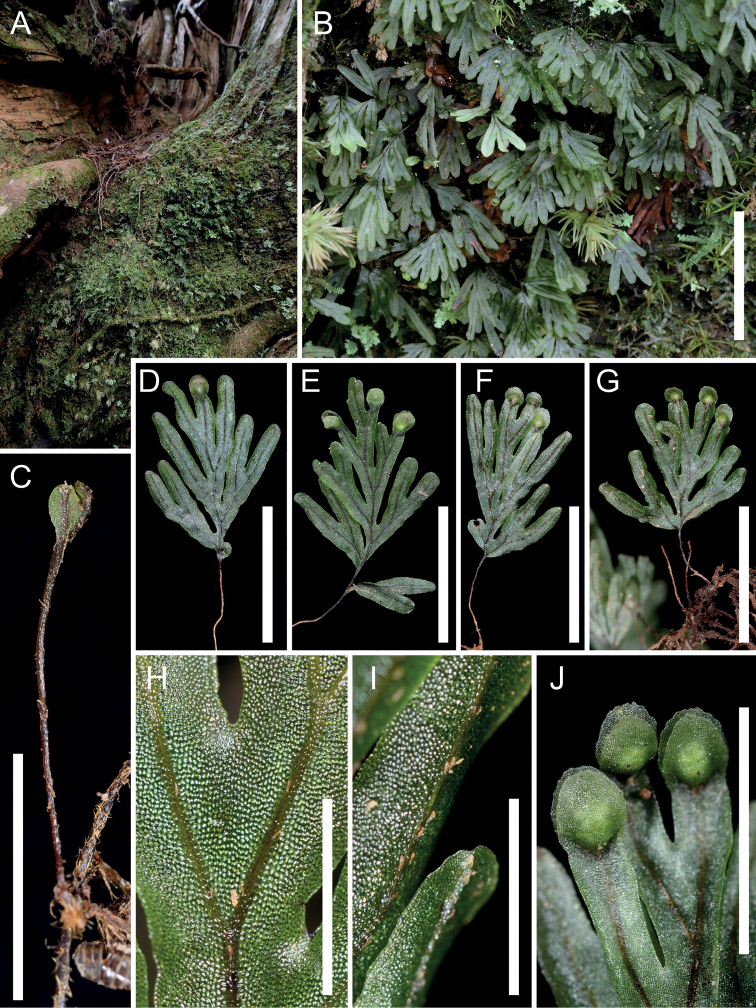
Habitat and morphology of *Hymenophyllumchamaecypericola*, from *Hsu 11888* (TAIF) **A, B** wild population growing on moss-covered basal trunk of a giant Chamaecyparisobtusavar.formosana**C** rhizome and young frond, showing the wingless and scarcely hairy stipe **D–G** fronds, adaxial views (**D, E**) and abaxial views (**F–G**) **H, I** laminae, adaxial view (**F**) and abaxial view (**G**), showing the minute yellow-brown clavate hairs on rachis and veins. J. Sori. Scale bars: 2 cm (**B**); 5 mm (**C, J**); 1 cm (**D–G**); 2 mm (**H, I**).

**Figure 2.**
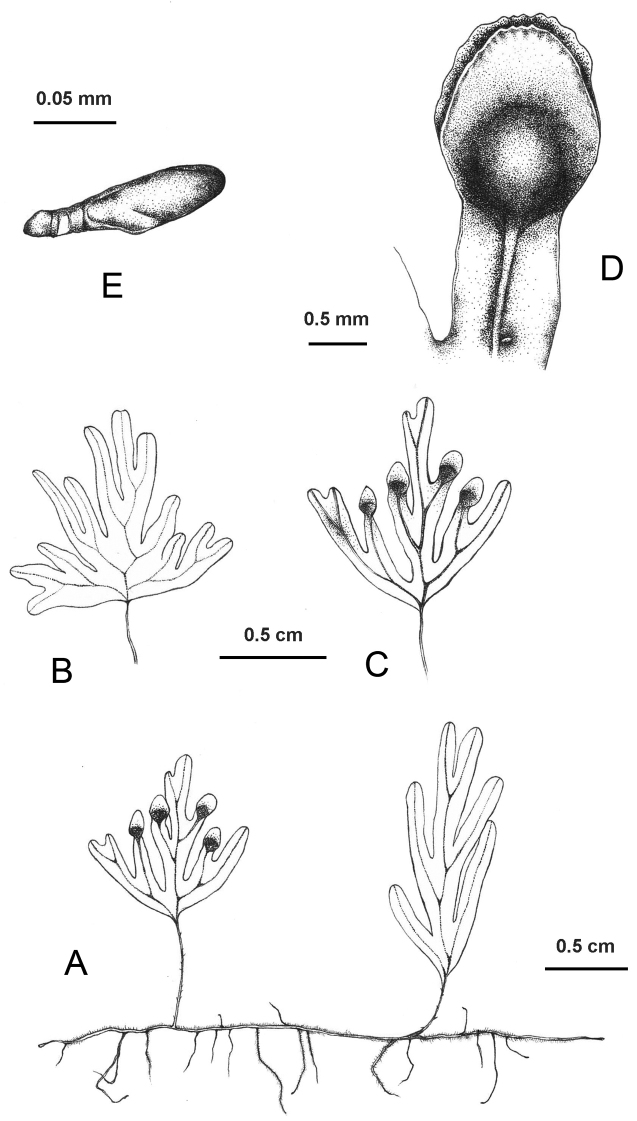
Line drawing of *Hymenophyllumchamaecypericola* T.C.Hsu & Z.X.Chang, sp. nov., based on the holotype *Z.X. Chang ZXC01438* (TAIF) **A** rhizome and fronds **B** sterile frond **C** fertile frond **D** sori **E** a clavate hair.

In 2019, the first author discovered a *Hymenophyllum* species with an uncertain assignment in a subtropical montane cloud forest of northern Taiwan. After observing its dwarf habit, superficially glabrous laminae, entire segments, and bivalvate, subentire involucres, we initially considered it to be a member belonging to subg. Mecodium, and tentatively identified it as *H.wrightii* Bosch, a small species distributed across East Asia and North America (Liu et al. 2013; Lee et al. 2014; Duffy et al. 2015). However, morphological distinctions between the specimen and *H.wrightii* were observed after careful investigation. This species produces apically distributed sori and minute clavate hairs along the rachis and costae (Figs 1, 2), which are absent in the subg. Mecodium, especially *H.wrightii*. In this study, we provided not only morphological but also phylogenetic evidence to circumscribe this uncertain species. With no recording of a similar species from previous literature, we determined it as a new species and have described it here as *Hymenophyllumchamaecyparicola* T.C.Hsu & Z.X.Chang, currently known to be endemic to Taiwan.

## ﻿Materials and methods

### ﻿Taxon sampling and molecular work

It total, we sampled 19 species, including most members of the East Asian subg. Hymenophyllum, all subg. Mecodium species in Taiwan, and *H.imbricatum* Blume from subg. Globosa as an outgroup (Hsu et al. 2019). This sampling also included three species from sect. Pseudomecodium, *H.exsertum* Wall. ex Hook., *H.oligosorum* and *H.pachydermicum* Ces., which was demonstrated to have frond characters similar to subg. Mecodium (Iwatsuki 1984, 1985). Their DNA was extracted using a modified CTAB protocol by Kuo (2015). Two chloroplast DNA (cpDNA) regions from these samples were sequenced: *rbcL* and *rps4-trnS* (*rps4* gene + *rps4-trnS* intergenic spacer). PCR reactions were each prepared in a 15 μL volume containing 20 ng genomic DNA, 1 × SuperRed PCR Master Mix RED (TOOLS, New Taipei City, Taiwan), and 0.5 μM of each primer. PCR products were then cleaned using ExoSAP-IT (Thermo Fisher Scientific, Waltham, Massachusetts, USA), and sequenced with ABI 3730XL (Thermo Fisher Scientific, Waltham, Massachusetts, USA) by the Genomics BioSci. & Tech. company (New Taipei City, Taiwan). Primers, voucher information and GenBank accession numbers are provided in the Appendices (Appendix 1 and Appendix 2).

### ﻿Phylogenetic analyses

In total, 49 sequences were used for analyses, including 23 newly generated ones from 13 samples and those used in Hsu et al. (2019) and Chen et al. (2021). These sequences were first aligned using MUSCLE (Edgar 2004) implemented in AliView (Larsson 2014), and the resulting alignments of the two cpDNA regions were then concatenated. Seven partitions were initially identified in the concatenated alignment, including each of three codon positions in *rbcL*, each of three codon positions in *rps4*, and *rps4-trnS* intergenic spacer. The best partition scheme and nucleotide substitution models were inferred by ModelFinder (Kalyaanamoorthy et al. 2017) based on AICc criteria, and applied for maximum likelihood (ML) and Bayesian phylogenetic analysis. IQ-TREE v. 1.6.10 (Nguyen et al. 2015) was used to reconstruct ML phylogeny in CIPRES (Miller et al. 2011) with 1000 standard bootstrap replicates. The Bayesian phylogenetic analysis was performed using MrBayes v.3.2.6 (Ronquist et al. 2012) in CIPRES (Miller et al. 2011) with two simultaneous runs and four chains. In each chain, 20 million generations were run, and sampled every 1000 generations. Tracer v. 1.7.1 (Rambaut and Drummond 2013) was used to determine the convergence through generations among chains. The first 25% of the generations was discarded as burn-in, and the posterior probabilities (PP) as branch supports of a Bayesian tree were then summarized.

## ﻿Results

**Figure 3.**
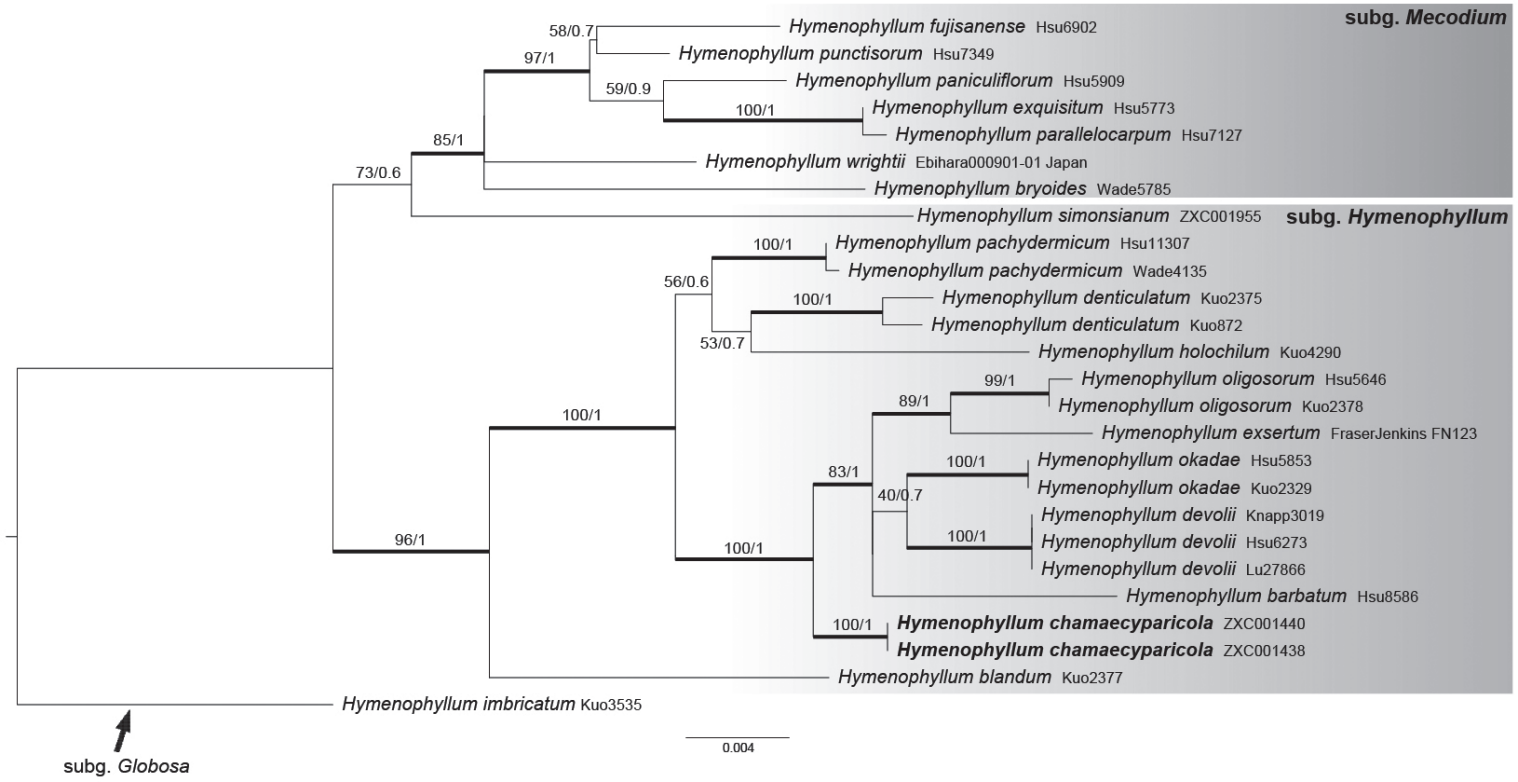
Maximum likelihood (ML) phylogeny of Hymenophyllumsubg.Hymenophyllum and Hymenophyllumsubg.Mecodium based on the chloroplast DNA dataset (*rbcL* + *rps4-trnS*). Branch support is indicated in ML bootstrap/ BI posterior probabilities.

The concatenated cpDNA dataset of *rbcL* (1365 bp) and *rps4-trnS* (1125 bp) contained a total of 2490 aligned sites. In our cpDNA phylogeny (Fig. 3), no conflicting relationship was found between ML and Bayesian trees. Our two samples of the uncertain *Hymenophyllum* (*H.chamaecyparicola* sp. nov.) possessed identical cpDNA sequences, and this species was found to be phylogenetically unrelated to *H.wrightii* in subg. Mecodium. Instead, it formed a strongly supported clade embedded in subg. Hymenophyllum and was well separated from all other species (Fig. 3). Interestingly, subg. Hymenophyllum as defined by Ebihara et al. (2006) was revealed to be non-monophyletic in our phylogenies because *H.simosianum* was found sister to subg. Mecodium with weak supporting values (BS/PP=73/0.6).

## ﻿Taxonomic treatment

### ﻿ Hymenophyllum chamaecyparicola

Taxon classificationPlantaeHymenophyllalesHymenophyllaceae

T.C.Hsu & Z.X.Chang sp. nov.

629D5B0D-BEC5-558C-A7DE-BF59BE24F86E

urn:lsid:ipni.org:names:77302867-1

Figs 1 , 2

#### Type.

Taiwan. Yilan County: Datong Township, Mingchih, 1200–1300 m, 31 January 2019, *Z.X. Chang ZXC01438* (holotype: TAIF; isotype: TAI).

#### Diagnosis.

Morphologically, *Hymenophyllumchamaecyparicola* is most similar to *H.wrightii* in sharing pinnate to bipinnatifid fronds, entire segment margins, and bivalvate, entire or subentire involucres. However, the new species could be clearly distinguished from *H.wrightii* by the presence of minute spathulate hairs on both surfaces of laminae (vs. glabrous laminae in *H.wrightii*) (Fig. 1H, I), the lack of two veinlets at the base of sori (vs. two veinlets at the base of sori in *H.wrightii*), and sori confined to apex or upper margins of laminae (vs. on short acroscopic segments close to rachis in *H.wrightii*) (Fig. 1J). This new species is phylogenetically related to *H.barbatum*, *H.devolii*, *H.exsertum*, *H.okadae* and *H.oligosorum*, while it could readily be distinguished from *H.barbatum*, *H.devolii* and *H.okadae* in having entire (vs. serrate) segment margins (Fig. 1D–G) and from *H.exsertum* and *H.oligosorum* in having pinnatifid to bipinnatifid (vs. bipinnatifid to tripinnatifid) laminae (Fig. 1D–G) sparsely covered with short (< 0.2 mm) clavate hairs (vs. densely covered with > 1 mm long acicular hairs) on abaxial surface of rachis and costae (Figs 1H, I, 2E).

#### Description.

Plants epiphytic. Rhizomes long creeping, blackish brown, 0.2–0.3 mm in diam, covered with caducous golden brown multicellular hairs, turning glabrescent when aged. Fronds (1)3–7(10) mm apart, (0.7)1–2.5(4.5) cm long, usually pendent. Stipes dark brownish, (1)2–12(25) mm long, ca. 0.15 mm in diam., wingless, with very sparse caducous hairs similar to those on the rhizomes, turning glabrescent when aged. Laminae pinnatifid to bipinnatifid, flabellate-orbicular, ovate or elliptic, (0.8)1–2.2(3.5) × (0.4)0.6–1.1(1.5) cm, membranous, base obtuse, apex rounded, with minute pale brownish clavate hairs along both surfaces of rachis, costae and veins, otherwise glabrous; clavate hairs up to 0.15 mm long, very sparse adaxially, sparse to scattered abaxially; rachises brown, slightly zigzag, winged throughout or sometimes wingless at base, wings up to ca. 0.2 mm wide, flat, entire; pinnae 2–4(5) pairs, alternate, forming acute angles with rachis, lower pinnae usually forked, rarely more dissected, upper pinnae usually simple, (2)3–8(11) mm long; ultimate segments oblong, (1)2–7(10) × 1.2–1.5 mm, apex rounded, entire, flat or slightly involute; veins simple, greenish brown, ending slightly below the apical margin. Sori 1–3(6) per lamina, confined to apex of lamina or sometimes scattered along upper margins, solitary and terminal on ultimate segments, segment lamina usually slightly constricted below sori; involucres bivalvate, orbicular, ovate-orbicular or elliptic, 1.2–2 × 1–1.5 mm, with a few minute clavate hairs at base, margins entire or minutely erose; receptacles inserted. Spores chlorophyllous, 64 per sporangium.

#### Additional specimen examined.

Taiwan. Yilan County: Datong Township, Mingchih, 1200–1300 m, 11 February 2019, *Chang ZXC01440* (TAIF); same loc., 11 July 2019, *Chang ZXC01670* (TAIF); same loc. and date, *Hsu 11888* (TAIF).

#### Distribution and habitat.

*Hymenophyllumchamaecyparicola* is endemic to Taiwan and currently known from scattered populations on a single ca. 2000 m^2^ mountain slope in *Chamaecyparis* montane mixed cloud forest (Li et al. 2013) around Mingchih (24.65361°N, 121.46950°E). It is epiphytic on bases of tree trunks and exposed roots of Chamaecyparisobtusavar.formosana (Hayata) Hayata.

#### Etymology.

The specific epithet, a noun in apposition, is derived from *Chamaecyparis*, a Gymnosperm genus, and –*cola*, dweller, alluding to unusual habitat of the new species occurring on the lower trunk of the giant C.obtusavar.formosana.

## ﻿Discussion

Our phylogeny generally agrees with the “modern” circumscriptions of Hymenophyllumsubg.Hymenophyllum and subg. Mecodium (Ebihara et al. 2006; Hennequin et al. 2010; Hsu et al. 2019; Vasques et al. 2019) with only one exception – *H.simonsianum*, which was placed within subg. Hymenophyllum based on morphology (Ebihara et al. 2006) but resolved here as an isolated lineage sister to subg. Mecodium (Fig. 3). Given that this unexpected position of *H.simonsianum* was weakly supported in our tree based on only two cpDNA regions, we consider that more sequence data is required to ascertain its systematic placement.

The phylogenetic position of *Hymenophyllumchamaecyparicola*, nested within subg. Hymenophyllum, was somewhat surprising in the beginning due to its superficial resemblance to *H.wrightii* in subg. Mecodium. However, after a detailed examination of the specimens, we concluded that its placement in subg. Hymenophyllum is also morphologically evident. Though hardly visible to the naked eye, *H.chamaecyparicola* bears clavate hairs on stipes and rachis, and such laminar trichomes are common in subg. Hymenophyllum but absent in subg. Mecodium (Ebihara et al. 2006; Hennequin et al. 2010; Hsu et al. 2019). Moreover, from our examination, two veinlets can be found at the bases of sori in members of subg. Mecodium sori but not in *H.chamaecyparicola* and other species of subg. Hymenophyllum as implied previously (Dubuisson et al. 2018).

Obviously, our sampling of Hymenophyllumsubg.Hymenophyllum (11 species), with an estimate of more than 100 species (Ebihara et al. 2006), remains insufficient. Even so, this study still provides some insights about interspecific relationships and systematics within this subgenus. We revealed for the first time that sect. Pseudomecodium, mainly defined by the combination of abaxially hairy veins and entire segments (Iwatsuki 1984, 1985), is non-monophyletic. In our tree, the four sampled species with entire segments, including *H.chamaecyparicola*, *H.exsertum*, *H.oligosorum* and *H.pachydermicum*, were placed in three different lineages (Fig. 3). In addition, our data strongly supported that *H.okadae*, recently reinstated from a synonym of *H.barbatum* based on a few subtle morphological characters (Knapp and Hsu 2017), is also phylogenetically distinct.

In addition to *H.chamaecyparicola*, *H.devolii* is another subg. Hymenophyllum species endemic to Taiwan. Our study then revealed that *H.devolii* is affiliated, not only morphologically but also phylogenetically, with its sympatric relatives, *H.okadae* and *H.barbatum*, which are also distributed in other East Asian regions. It will be very worthy to further study the speciation pathways behind these endemic ferns in Taiwan. A comprehensive sampling in the subgenus, especially from Southeast Asia, and a dated phylogeny are ultimately necessary to clarify the evolutionary history of these Taiwan endemic ferns.

### ﻿Key to subg. Hymenophyllum and *Mecodium* species in Taiwan

**Table:** 

1	Laminae glabrous, indumentum absent along the stipes, rachises, and veins	**2**
–	Indumentum present along the stipes, rachises, and veins	**6**
2	Stipes wingless or only with decurrent wings at apexes	**3**
–	Stipes narrowly winged to base or at least to middle	**4**
3	Stipes reddish brown, wingless; involucres orbicular, distinctly wider than joint segments	***H.punctisorum***
–	Stipes dark brownish, only with decurrent wings at apexes; involucres ovate-orbicular or ovate, roughly as wide as joint segments	***H.parallelocarpum***
4	Laminae shorter than 6 cm; sori densely aggregated at lamina apexes	***H.paniculiflorum***
–	Laminae variable; sori never densely aggregated at lamina apexes	**5**
5	Rachis and costa wings weakly crispate or flat; ultimate segments nearly flat; involucres ovate to ovate-triangular	***H.fujisanense***
–	Rachis and costa wings strongly crispate; ultimate segments contorted; involucres oval to suborbicular	***H.exquisitum***
6	Segment margins entire	**7**
–	Segment margins serrate	**8**
7	Laminae pinnatifid to bipinnatifid; minute pale brownish clavate hairs (ca. < 0.2 mm) present on both surfaces of rachises and veins	***H* . *chamaecyparicola***
–	Laminae bipinnate to tripinnatifid; brownish setae (ca. > 1 mm) present on both surfaces of rachises and veins	***H.oligosorum***
8	Involucres obconic-tubular; receptacles exserted	**9**
–	Involucres cleft to base, not obconic-tubular; receptacles included in involucres	**11**
9	Stipes and rachises wingless; involucres serrate at apexes	***H* . *blandum***
–	Stipes and rachises winged; involucres entire or toothed at apexes	**10**
10	Laminae crispate; involucres toothed; spine-like protrusions present on base of involucres	***H* . *denticulatum***
–	Laminae flat; involucres entire; spine-like protrusions absent on base of involucres	***H* . *holochilum***
11	Involucres orbicular to ovate	**12**
–	Involucres oblong to oval	**13**
12	Rachis wings involute; involucres orbicular to oblate, dentate at apexes	***H* . *okadae***
–	Rachis wings recurved to revolute; involucres orbicular to ovate, entire or sometimes slightly crenate at apexes	***H* . *devolii***
13	Laminae ovate; segments 2 mm broad; costae of sterile pinna with more than 2 pairs of costules	***H* . *barbatum***
–	Laminae linear-oblong to linear-lanceolate; segments 2–4 mm broad; costae of sterile pinna only with 1 or 2 pair of costules	***H* . *simonsianum***

## Supplementary Material

XML Treatment for Hymenophyllum chamaecyparicola

## ﻿Appendix 1

**Table A1.** Voucher and sequence information of *Hymenophyllum* species for the phylogenetic analyses. GenBank accessions (*rps4- trnS* and *rbcL*) are under their columns, respectively. The symbol “–” means not available; the symbol “* ” means newly generated sequences in this study.

Taxon	Voucher specimen number	Collection locality	Herbarium	*rps4-trnS*	*rbcL*
*H.barbatum*	*Hsu 8586*	Taiwan (Taoyuan County)	TAIF	ON773153*	ON652817*
*H.blandum*	*Kuo 2377*	Taiwan (Yilan County)	TAIF	ON773147*	ON773829*
*H.bryoides*	*Wade 5785*	Vietnam	TAIF	MW478759	MW478758
*H.chamaecyparicola*	*ZXC001440*	Taiwan (Yilan County)	TAIF	ON773148*	ON773830*
*H.chamaecyparicola*	*ZXC001438*	Taiwan (Yilan County)	TAIF	ON773149*	ON773831*
*H.denticulatum*	*Kuo 872*	Taiwan (Pingtung County)	TAIF	ON773146*	ON773828*
*H.denticulatum*	*Kuo 2375*	Taiwan (Yilan County)	TAIF	–	ON773827*
*H.devolii*	*Knapp 3019*	Taiwan (Taitung County)	P	MF144616	MF144660
*H.devolii*	*Lu 27866*	Taiwan (Taitung County)	TAIF	–	ON773833*
*H.devolii*	*Hsu 6273*	Taiwan (Taitung County)	TAIF	MN266569	MN266660
*H.exquistum*	*Hsu 5773*	Taiwan (Hsinchu County)	TAIF	MH211098	MH211069
*H.exsertum*	*Fraser-Jenkins-FN123*	India	TAIF	ON773154 *	ON773836*
*H.fujisanense*	*Hsu 6902*	Taiwan (Taitung County)	TAIF	MH211087	MH211058
*H.holochilum*	*Kuo 4290*	Taiwan (Pingtung County)	TAIF	MH265124	MH265124
*H.imbricatum*	*Kuo 3535*	Philippines	TAIF	MH211105	MH211076
*H.okadae*	*Hsu 5853*	Taiwan (Taoyuan County)	TAIF	MH211103	MH211074
*H.okadae*	*Kuo 2329*	Taiwan (Yilan County)	TAIF	ON773145*	ON773826*
*H.oligosorum*	*Kuo 2378*	Taiwan (Yilan County)	TAIF	–	ON773825*
*H.oligosorum*	*Hsu 5646*	Taiwan (Hualien County)	TAIF	MH211102	MH211073
*H.pachydermicum*	*Hsu 11307*	Vietnam	TAIF	ON773151*	ON773834*
*H.pachydermicum*	*Wade 4135*	Vietnam	TAIF	ON773152*	ON773835*
*H.paniculiflorum*	*Hsu 5909*	Taiwan (Taichung County)	TAIF	MH211097	MH211068
*H.parallelocarpum*	*Hsu 7127*	Taiwan (Pingtung County)	TAIF	MH211101	MH211072
*H.punctisorum*	*Hsu 7349*	Taiwan (Nantou County)	TAIF	MH211083	MH211054
*H.simonsianum*	*ZXC001955*	Taiwan (Nantou County)	TAIF	ON773150*	ON773832*
*H.wrightii*	*Ebihara000901-01*	Japan	TI	AY775430	AB083277

## ﻿Appendix 2

**Table A2.** Primers used in this study.

Name	Region	Sequence (5’-3’)	Reference
aF	*rbcL*	ATGTCACCACAAACAGAGACTAAAGC	Hasebe et al. 1994
1379R	*rbcL*	TCACAAGCAGCAGCTAGTTCAGGACTC	Pryer et al. 2001
Fern rbcL fVGF	*rbcL*	GAGACTAAAGCAGGTGTTGGATTCA	This study
Fern rbcL rVVG	*rbcL*	GTTCCCCYTCTAGTTTRCCTACTAC	This study
rps5	*rps4-trnS*	ATGTCCCGTTATCGAGGACCT	Nadot et al. 1994
trnS	*rps4-trnS*	TACCGAGGGTTCGAATC	Souza-Chies et al. 1997
